# Epidemiology of Cigarette and Smokeless Tobacco Use among South Asian Immigrants in the Northeastern United States

**DOI:** 10.1155/2011/252675

**Published:** 2011-05-17

**Authors:** Cristine D. Delnevo, Michael B. Steinberg, Shawna V. Hudson, Rajiv Ulpe, Robert S. DiPaola

**Affiliations:** ^1^UMDNJ-School of Public Health and The Cancer Institute of New Jersey, 335 George Street, Suite 2100, New Brunswick, NJ 08903, USA; ^2^UMDNJ-Robert Wood Johnson Medical School and The Cancer Institute of New Jersey, 125 Paterson Street, Room 2300, New Brunswick, NJ 08903, USA; ^3^UMDNJ-Robert Wood Johnson Medical School and The Cancer Institute of New Jersey, 195 Little Albany Street, Room 5569, New Brunswick, NJ 08903-2681, USA; ^4^UMDNJ-School of Public Health, 335 George Street, Suite 2100, New Brunswick, NJ 08903, USA; ^5^UMDNJ-Robert Wood Johnson Medical School and The Cancer Institute of New Jersey, 195 Little Albany Street, Room 2002B, New Brunswick, NJ 08903-2681, USA

## Abstract

As the most preventable cause of death in the world today, understanding tobacco use among one of the fastest growing ethnic/racial groups is warranted. We explore cigarette and smokeless tobacco (SLT) use among South Asians in NJ and the Northeast using the Tobacco Use Supplement to the Current Population Survey. Overall, tobacco use rates among South Asians were similar or lower than the population. However, in NJ, South Asian males had the highest SLT rate (2.7%) and in the Northeast, White (AOR = 5.8, 95%  CI = 3.7–9.4) and South Asian males (AOR = 4.0, 95%  CI = 1.5–10.6) had significantly higher odds of current SLT use relative to non-White males. Tobacco use among South Asians was not homogeneous; Pakistanis are overrepresented among cigarette smokers while Indians are overrepresented among SLT users. Given the differential tobacco use among and within South Asian, disaggregating data to understand tobacco use behaviors is necessary to develop effective interventions for tobacco cessation.

## 1. Introduction

Tobacco is the single most preventable cause of death in the world today, including South Asian countries like India where there are disparities in chronic diseases like cancer and cardiovascular disease that have surpassed infectious disease as the leading causes of death. South Asians are the third largest Asian group in the United States, comprising 1.89 million people and are among the fastest growing racial groups in New Jersey and the Northeast [1]. In 2000, one out of three South Asians reside in the Northeast, and there were almost 170,000 South Asians living in New Jersey, representing the 3rd largest statewide South Asian population in the country with the large majority of South Asian immigrants coming from India, Pakistan, Bangladesh, and Sri Lanka [1].

Despite marked health disparities in South Asians internationally compared to the US population (e.g., cancer, heart disease, and diabetes) [2–4], little is known about the health status of South Asians residing in the US Paradoxically, the South Asian population in the US is generally viewed as a successful immigrant group, resulting in a characterization known as the “Model Minority Myth.” This concept describes a minority ethnic, racial, or religious group whose members achieve a higher degree of success, affluence, and thus good health, than the population as a whole. However, recent data strongly contradict the notion that South Asians are uniformly affluent and healthy and highlight the growing heterogeneity of this group [1, 2, 5, 6].

Indeed, India is the second largest consumer of tobacco in the world, and national data indicate that 47% of men and 14% of women either smoke or chew tobacco [7]. Likewise, nearly one out of three adults in Bangladesh use some form of tobacco [8], and one out of three Pakistani males use tobacco daily [9]. Studies conducted in the UK suggest that South Asians who immigrate may have lower rates of smoking overall than the general population [10, 11]. However, other studies have found high rates of smoking in certain subgroups of South Asians, particularly Bangladeshis [12, 13]. Studies of tobacco use in South Asian immigrants in the US are limited and not applicable to the general population for two main reasons. First, studies have been geographically limited to a community, city, or single state [14–17]. Second, despite the traditional role of smokeless tobacco in South Asian cultures, some studies have focused only on cigarette smoking [2, 16, 17]. 

The paucity of research on tobacco use behaviors in South Asians is due in part to the fact that despite a distinct cultural and geographical background, South Asians are almost always aggregated into a broad “Asian” category, thus potentially masking subgroup differences and preventing identification of potential health disparities between subgroups. However, given that the explosive growth in the South Asian population is fairly recent and largely attributed to immigration, it is methodologically possible to identify South Asians in the Tobacco Use Supplement to the Current Population Survey (CPS), as the survey collects country of origin. The current study is the first to use population level behavioral surveillance data to explore patterns of cigarette and smokeless tobacco use among South Asians residing in New Jersey and the Northeast US and to explore tobacco use behavior by country of origin.

## 2. Methods

### 2.1. Data Source

We analyzed New Jersey and Northeast specific data from the 2003 and the 2006/7 Tobacco Use Supplement to the Current Population Survey (TUS-CPS). The details of the TUS-CPS sampling design and data collection methods are provided elsewhere [18]. In brief, the TUS-CPS uses an area probability sampling design to select a stratified probability sample of clusters of households. Approximately 56,000 households are surveyed in a given month using computer-assisted telephone interviewing (CATI) and computer-assisted personal interviewing (CAPI) methods. State estimates may be generated from the national TUS by combining multiple months of data. Individual level self-response rates for the TUS-CPS questionnaire are approximately 65–72% for those households completing the basic CPS household survey (response rates range from 93 to 97%). To increase sample size, we merged data from 2003 and 2006/7 for our analyses. The overall sample size for New Jersey was 7,354, of which 176 were South Asian, and for the Northeast there were 71,152 total cases of which 583 were South Asian.

### 2.2. Race/Ethnicity, Country of Origin, and Immigrant Status Measures

We constructed a single, five-level variable for race/ethnicity which included White, Black, Hispanic, Asian, and South Asian. This was constructed from five survey questions: race, Hispanic origin, country of origin, mother's country of origin, and father's country of origin. South Asians were defined as those individuals who indicated that their country of origin or the country of origin for one of their parents were from India, Pakistan, or Bangladesh. While the TUS did not permit the identification of other South Asian countries of origin (e.g., Sri Lanka, Nepal), the three countries we could identify comprise 98.5% of South Asian immigrants in the US [1]. In addition, we created an additional variable for South Asians only that reflected their immigration status and country of origin which resulted in four mutually exclusive categories: first generation American of South Asian descent (i.e., born in the US, but at least one parent is from India, Pakistan, or Bangladesh), immigrant from India, immigrant from Pakistan, and immigrant from Bangladesh.

### 2.3. Tobacco Measures

We applied standard tobacco surveillance criteria for calculating adult tobacco use prevalence [19]. Our measures of cigarette smoking were derived from three questions resulting in two measures: ever smoker (i.e., smoked 100 cigarettes in their lifetime), and current smoker (i.e., smoked 100 cigarettes and now smokes everyday or some days). With respect to smokeless tobacco, the TUS-CPS does not include a lifetime threshold question (e.g., smoked 100 cigarettes) for smokeless tobacco use, but does inquire about snuff tobacco and chew tobacco separately. Our measures of smokeless tobacco use were derived from four questions resulting in two measures: ever smokeless user (i.e., has used snuff or chew), and current smokeless user (i.e., now uses snuff or chew everyday or some days).

### 2.4. Analysis

Sample replicate weights were applied to adjust for nonresponse and the varying probabilities of selection, including those resulting from oversampling, providing results representative of New Jersey and the Northeast's adult population. SUDAAN statistical software, which corrects for the complex sample design, was utilized to generate point estimates and adjusted odds ratios (AOR) with 95% confidence intervals [20].

## 3. Results

Overall, 74.8% of adults in the Northeast were White, 10.5% were Black, 9.7% were Hispanic, 3.6% were Asian/PI (not South Asian descent), and 1.3% were South Asian. As shown in Table 1, South Asians are demographically different than their White, Black, Hispanic, and Asian counterparts. First, South Asians were more likely to be male (60.0%) compared to all other racial/ethnic groups and to the overall sample (47.5%). With respect to age, South Asians had a lower proportion of adults over the age of 65 compared to Whites, Blacks, and Asians. South Asians also had the largest proportion of adults with at least a college education (70.8%); this is more than twice the rate of Whites overall, and three to four times the rate of Black and Hispanic adults. Lastly, within the Northeast, South Asians are over-represented in New Jersey with 34.8% residing there. 

Table 2 summarizes the prevalence of current and ever cigarette and smokeless tobacco use in New Jersey and in the US Northeast by race/ethnicity and gender. Overall, 16.9% of males in New Jersey report currently smoking cigarettes and South Asians had current smoking rates (12.0%) below their other racial/ethnic counterparts. This pattern is consistent in the Northeast. In general, South Asian females had low rates of cigarette smoking both in New Jersey and the Northeast. 

Smokeless tobacco use is predominately a white male behavior in the US, yet in NJ, South Asian males (2.7%) have the highest rates of current use among males. In the northeast, South Asian males (1.4%) currently use smokeless tobacco at a rate somewhat lower, but not significantly different from White males (2.3%). These rates are somewhat confounded by the different ages and educational status of South Asians, as smokeless tobacco use is more common among younger adults and those with lower levels of education. When education and age are adjusted for in a logistic regression, white males (AOR = 5.8, 95% CI = 3.7–9.4) and South Asian males (AOR = 4.0, 95% CI = 1.5–10.6) had significantly higher odds of current smokeless tobacco use relative to non-White males. Rates of smokeless use are extremely low among females of all racial/ethnic groups both in New Jersey and the Northeast. However, ever smokeless use is notable among South Asian females in New Jersey (1.7%) compared to females overall in New Jersey (0.2%). 

Tobacco use behavior among South Asians is not homogeneous (see Table 3). Overall, in the Northeast, while Pakistanis make up only 9.6% of all South Asian males, they are overrepresented among current cigarette smokers, but not ever smokers, raising questions about cessation. Indeed, the ever smoking rate among Pakistani males in the northeast is 24.3%, and the current smoking rate is 22.4%, suggesting that few male Pakistani ever smokers have quit. On the other hand, Indian males who make up 70% of all South Asians, comprise 85.9% of the current smokeless tobacco users. Lastly, the data suggest a possible acculturation effect among females. Indeed, while first generation female Americans of South Asian descent comprise 17.6% of South Asian females overall, they are overrepresented among South Asian female cigarette smokers (50.0%).

## 4. Discussion

This study represents one of the only descriptions of tobacco use by South Asians in the United States at the population level. Despite common misperceptions regarding health behaviors and status, South Asians in this study demonstrate important tobacco-use behaviors including lower quit rates, high rates of smokeless tobacco use, and significant heterogeneity regarding these behaviors. Our data support the existing literature demonstrating lower rates of cigarette use in South Asians than other racial/ethnic groups [10, 11]. Similar to the prior data by Choi et al. [21], our study supports the hypothesis that acculturation has a beneficial effect in Asian American men and harmful effects on women and adolescents. 

Despite having lower rates of ever and current cigarette smoking than other racial/ethnic groups, it is important to recognize cigarette smoking behavior differed by country of origin among South Asian males, and the data suggest that Pakistani males who have ever smoked cigarettes continue to use tobacco. This may be partially explained by the age distribution of South Asians, who tended to be younger; a group that is less likely to quit than older ever smokers. However, the findings do raise concern that South Asian immigrants who smoke may be less motivated to quit and/or have a more difficult time stopping smoking. This possibility is supported in the, albeit limited, research literature. In the UK, the intention of South Asian males to give up smoking was similar to the general population; however, actual quit rates were much lower, and utilization of cessation services was lower among South Asians [13]. Reasons for this are unclear. Bush et al. [12] suggested that the social acceptability of smoking in Pakistani and Bangladeshi communities may contribute to a lower quit rate while White et al. [22] noted a low level of awareness of the health risks associated with smoking and insufficient use of professional advice/smoking cessation aids among this population. 

Smokeless tobacco use is an especially important behavior among certain South Asians, especially males and those from India. While in the US, smokeless tobacco refers to moist snuff or chewing tobacco, the term “smokeless tobacco” is broad and refers to over 30 different types of products including those indigenous smokeless tobacco products that are most frequently used in South Asia, including but not limited to paan, paan masala, zarda, betel quid with tobacco, and gutka [23–25]. Health effects linked to smokeless tobacco use in general include oral cancer, pancreatic cancer, oral diseases such as periodontal diseases, precancerous lesions, and risk factors for cardiovascular diseases, diabetes, reproductive health effects, and overall mortality [25]. Moreover, there is conclusive evidence that betel quid chewed with and without tobacco, tobacco with lime, and other tobacco mixtures specific to South Asian smokeless tobacco products increase the risk of oral cancer [24]. Not surprisingly, data indicated that oral cancer incidence and mortality among people of South Asian descent are almost twice those of global rates [26] and are largely attributed to the use of indigenous tobacco products [27]. Data from the UK, Canada, and California suggest that South Asian immigrants may maintain these higher rates of oral cancer compared to general population [28–31]. Given the growth in this population, future cancer surveillance is clearly warranted. 

Finally, the heterogeneity of this sample of South Asians demonstrates that generalization of tobacco surveillance findings can lead to erroneous conclusions. For example, this group of South Asians illustrates high rates of cigarette smoking among Pakistanis while Indians represent most smokeless tobacco users. While studies conducted in the UK and the US have suggested that South Asians in aggregate may have lower rates of smoking than the general population [10, 11], other studies have found high rates of smoking in certain subgroups of South Asians, particularly Bangladeshis [12, 13]. Therefore, population level data collection measures that consider South Asians as a single group will likely miss important country of origin differences in tobacco use behavior, and possibly oral cancer rates. This has critical tobacco dependence treatment implications as effective treatments may vary based on the particular tobacco product. 

This study has some limitations that bear mentioning. First, we limited our focus to the northeast. While one out of three South Asians reside in the northeast, the extent to which the findings reported here are generalizable to those residing elsewhere is a valid concern. However, we could find no published reports which provided details on the extent to which those in the northeast may differ from their other US counterparts. Second, the number of participants, especially females and those from particular countries of origin are limited. Therefore, conclusions based on these small numbers should be made with caution. However, a strength of this study was our ability to identify and disaggregate South Asians from Asians overall. Our initial analysis (not shown in this paper) indicated that had we analyzed Asians in aggregate, the rates of smokeless tobacco use would have been masked, yielding extremely low prevalence estimates. Third, tobacco control surveillance systems, which are population-based, ask about traditional “Western” tobacco products, such as cigarettes and moist snuff. Subsequently, we suspect that the prevalence data presented here may underestimate tobacco use as indigenous tobacco products used by South Asians, such as bidi cigarettes, as well as gutka, zarda and paan masala, are not addressed on these surveys. Moreover, some of these indigenous smokeless tobacco products have high levels of tobacco-specific nitrosamines (TSNA) and are associated with substantial health risks that may be greater than their Western counterparts [32]. Lastly, methodological limitations in the TUS-CPS with regards to country of origin restrict our ability to further explore important within group variation for cigarette smoking and smokeless tobacco use among other South Asian immigrant populations (e.g., Sri Lanka, Nepal). 

Despite these limitations, this study provides important population level data about differential tobacco use and emphasizes the need for further research that disaggregates South Asian populations. Study findings also point to the need to develop, test, and disseminate multiple, targeted tobacco cessation and treatment interventions that take into account important sociocultural differences among South Asian populations as well as differences based on the particular tobacco product used. While only three empirical studies investigating smoking cessation interventions targeting the broader category of Asian Americans have been documented in the literature, the findings suggest that scientifically valid, culturally tailored, and language-specific interventions are effective in reducing tobacco usage among ethnically specific Asian American populations [33]. Lastly, the use of indigenous smokeless tobacco among South Asians deserves attention in the context of the current “harm reduction” debate, where some tobacco control professionals argue that smokers should switch to smokeless tobacco if they cannot quit. This debate is largely focused around “snus” a very low tobacco-specific nitrosamine (TSNA) product with notably lower health risks than cigarettes. The data available regarding indigenous South Asian SLT products are highly varied with the International Agency for Research on Cancer (IARC) finding higher levels of some TSNA (e.g., NNK) in the smokeless products used in India relative to North American and European smokeless tobacco products [24, 25], and since levels of TSNA's are influenced by many factors (e.g., fermentation, processing, other nontobacco carcinogens such as areca nut), these products may be associated with substantially greater health risks than some Western products. For this reason, even though more data are needed describing the health risks and carcinogenic potential of South Asian SLT products that are available in the US, what is clear is that they are certainly not without harm and should not be marketed to the South Asian community as a safe alternative to smoking.

## Figures and Tables

**Table 1:** Demographic characteristics among adults in the US northeast by race/ethnicity, 2003–2006/7 Tobacco Use Supplement to the Current Population Survey.

	White		Black		Hispanic		Asian		South Asian		Overall	
(Unweighted *n*)	*n* = 60,505		*n* = 4,449		*n* = 4,485		*n* = 1,442		*n* = 585		*n* = 71,466	
	%	95% CI	%	95% CI	%	95% CI	%	95% CI	%	95% CI	%	95% CI
*Gender*												
Male	47.7	(47.5–47.9)	44.0	(43.3–44.6)	48.1	(46.7–49.4)	46.3	(44.0–48.6)	59.9	(56.2–63.7)	47.5	(47.4–47.6)
Female	52.3	(52.1–52.5)	56.0	(55.4–56.7)	51.9	(50.6–53.3)	53.7	(51.4–56.0)	40.1	(36.3–43.8)	52.5	(52.4–52.6)
*Age group*												
18–24	9.6	(9.2–9.9)	14.8	(13.6–16.1)	15.6	(14.3–16.9)	10.8	(8.9–12.7)	9.6	(6.2–13.0)	10.7	(10.4–11.0)
25–44	35.6	(35.2–36.0)	41.5	(40.2–42.8)	50.0	(48.4–51.6)	48.5	(45.7–51.2)	59.9	(54.1–65.6)	38.4	(38.1–38.7)
45–64	34.7	(34.2–35.1)	30.0	(29.0–31.0)	26.2	(24.9–27.6)	29.4	(26.9–31.8)	24.3	(20.2–28.4)	33.0	(32.7–33.4)
65+	20.2	(19.8–20.6)	13.7	(12.7–14.6)	8.2	(7.2–9.3)	11.4	(9.1–13.7)	6.3	(3.8–8.7)	17.8	(17.5–18.2)
*Education*												
Less than HS	9.4	(9.0–9.7)	19.4	(17.9–20.9)	36.5	(34.5–38.5)	12.0	(9.7–14.3)	5.1	(2.3–7.8)	13.1	(12.7–13.5)
HS	32.9	(32.3–33.5)	34.6	(33.0–36.2)	30.0	(28.5–31.6)	21.0	(18.3–23.8)	14.2	(10.4–18.0)	32.1	(31.6–32.7)
Some college	23.6	(23.1–24.1)	26.1	(24.7–27.6)	19.3	(18.0–20.7)	15.6	(13.5–17.7)	10.0	(6.8–13.2)	23.0	(22.6–23.4)
College	34.1	(33.3–34.8)	19.9	(18.5–21.3)	14.1	(12.8–15.5)	51.4	(47.6–55.1)	70.8	(65.6–75.9)	31.8	(31.1–32.4)
*State*												
Connecticut	6.6	(6.5–6.8)	5.1	(4.7–5.4)	5.8	(4.8–6.7)	3.7	(2.7–4.7)	8.1	(4.9–11.3)	6.3	(6.3–6.3)
Maine	3.2	(3.2–3.2)	0.2	(0.1–0.2)	0.2	(0.1–0.3)	0.4	(0.3–0.6)	0.2	(0.0–0.3)	2.4	(2.4–2.5)
Massachusetts	13.4	(13.1–13.6)	5.9	(5.4–6.3)	7.9	(6.4–9.4)	11.6	(8.6–14.5)	7.7	(4.7–10.7)	11.9	(11.8–12.0)
New Hampshire	3.0	(3.0–3.1)	0.2	(0.1–0.2)	0.4	(0.3–0.6)	1.1	(0.6–1.5)	1.1	(0.5–1.7)	2.4	(2.4–2.4)
New Jersey	13.8	(13.5–14.1)	19.0	(18.2–19.8)	21.9	(19.9–23.8)	21.6	(18.4–24.7)	34.8	(27.7–41.9)	15.7	(15.6–15.7)
New York	30.2	(29.8–30.6)	48.8	(47.7–49.9)	54.4	(52.0–56.8)	51.9	(47.5–56.2)	37.7	(31.2–44.3)	35.4	(35.3–35.5)
Pennsylvania	26.1	(25.9–26.4)	20.0	(19.3–20.8)	7.3	(5.9–8.7)	8.3	(5.9–10.7)	9.6	(6.3–13.0)	22.8	(22.7–22.9)
Rhode Island	2.2	(2.1–2.2)	0.8	(0.7–0.9)	2.1	(1.7–2.4)	1.3	(0.9–1.7)	0.5	(0.1–0.8)	2.0	(2.0–2.0)
Vermont	1.5	(1.5–1.5)	0.1	(0.1–0.1)	0.1	(0.1–0.1)	0.2	(0.1–0.3)	0.3	(0.1–0.5)	1.2	(1.2–1.2)

**Table 2:** Prevalence* of ever and current cigarette and smokeless tobacco use among adults in New Jersey and the US northeast by gender and race/ethnicity, 2003–2006/7 Tobacco Use Supplement to the Current Population Survey.

	New Jersey								Northeast							
	Males				Females				Males				Females			
			Smokeless tobacco				Smokeless tobacco				Smokeless tobacco				Smokeless Tobacco	
	Cigarette use		use		Cigarette use		use		Cigarette use		use		Cigarette use		use	
	%	95% CI	%	95% CI	%	95% CI	%	95% CI	%	95% CI	%	95% CI	%	95% CI	%	95% CI
*White*																
Ever	45.9	(43.8–48.0)	8.7	(7.5–9.9)	39.2	(37.2–41.2)	0.3	(0.3–0.9)	47.4	(46.5–48.2)	13.4	(12.7–14.0)	40.4	(39.7–41.1)	1.1	(1.0–1.2)
Current	16.1	(14.4–17.8)	1.0	(0.5–1.5)	13.5	(12.2–14.8)	0.1	(0.0–0.1)	19.2	(18.6–19.8)	2.3	(2.0–2.6)	16.7	(16.2–17.2)	0.0	(0.0–0.0)
*Black*																
Ever	36.8	(30.4–43.3)	4.2	(1.8–6.7)	26.5	(22.0–31.0)	0.6	(0.0–1.2)	35.9	(33.1–38.8)	3.6	(2.5–4.6)	27.0	(25.1–29.0)	0.8	(0.4–1.1)
Current	20.6	(14.8–26.3)	0.3	(0.0–0.8)	14.9	(11.4–18.4)	0.0	(0.0–0.0)	20.5	(18.0–23.0)	0.5	(0.1–0.9)	15.7	(14.0–17.4)	0.2	(0.1–0.3)
*Hispanic*																
Ever	31.7	(26.5–37.0)	2.7	(1.1–4.3)	17.1	(13.4–20.7)	0.4	(0.0–0.6)	32.0	(29.6–34.4)	2.9	(2.1–3.6)	20.6	(18.9–22.2)	0.3	(0.1–0.4)
Current	18.3	(14.1–22.5)	0.0	(0.0–0.0)	8.9	(5.8–12.0)	0.0	(0.0–0.0)	18.9	(16.9–20.8)	0.5	(0.1–0.8)	11.9	(10.5–13.2)	0.0	(0.0–0.0)
*Asian*																
Ever	36.7	(27.8–45.6)	0.0	(0.0–0.0)	3.3	(0.3–6.2)	0.0	(0.0–0.0)	28.5	(24.9–32.1)	2.0	(0.8–3.1)	7.9	(5.7–10.1)	0.1	(0.1–0.3)
Current	18.6	(10.9–26.3)	0.0	(0.0–0.0)	1.3	(0.0–3.2)	0.0	(0.0–0.0)	15.9	(12.7–19.1)	0.4	(0.1–0.9)	4.6	(2.8–6.4)	0.0	(0.0–0.0)
*South Asian*																
Ever	30.9	(21.3–40.6)	2.7	(0.0–6.0)	3.6	(0.0–7.7)	1.7	(0.0–2.6)	22.7	(17.6–27.7)	2.5	(0.8–4.1)	4.3	(1.5–7.0)	0.7	(0.1–1.5)
Current	12.0	(5.4–18.6)	2.7	(0.0–6.0)	3.6	(0.0–7.7)	0.0	(0.0–0.0)	9.5	(5.7–13.2)	1.4	(0.2–2.6)	2.6	(0.2–5.0)	0.0	(0.0–0.0)
*Overall*																
Ever	41.8	(40.0–43.6)	6.7	(5.8–7.6)	31.9	(30.2–33.6)	0.2	(0.3–0.7)	43.7	(42.9–44.4)	10.8	(10.3–11.3)	35.5	(34.8–36.1)	0.9	(0.8–1.0)
Current	16.9	(15.4–18.4)	0.8	(0.4–1.1)	12.2	(11.1–13.4)	0.1	(0.0–0.1)	19.0	(18.4–19.6)	1.9	(1.6–2.1)	15.5	(15.0–16.0)	0.0	(0.0–0.1)

*Weighted percentages.

**Table 3:** Country of origin and immigration status among South Asian adults in the US northeast overall and among ever and current cigarette smokers and smokeless tobacco users by gender, 2003–2006/7 Tobacco Use Supplement to the Current Population Survey.

	Overall	Ever smoker	Current smoker	Ever SLT	Current SLT
*Males*					
1st gen American of South Asian descent	8.4	10.6	6.0	1.3	0.0
India	70.0	66.1	57.8	90.8	85.9
Pakistan	9.6	10.7	23.4	7.9	14.1
Bangladesh	12.0	12.5	12.9	0.0	0.0

*Females*					
1st gen American of South Asian descent	17.6	46.6	50.0	31.2	No current use
India	65.6	43.9	34.4	68.8	
Pakistan	11.1	9.5	15.5	0.0	
Bangladesh	5.6	0.0	0.0	0.0	
